# Common SNP rs6564851 in the *BCO1* Gene Affects the Circulating Levels of β-Carotene and the Daily Intake of Carotenoids in Healthy Japanese Women

**DOI:** 10.1371/journal.pone.0168857

**Published:** 2016-12-22

**Authors:** Suemi Yabuta, Masanori Urata, Roseline Yap Wai Kun, Motofumi Masaki, Yoshihiro Shidoji

**Affiliations:** 1 Molecular and Cellular Biology, Graduate School of Human Health Science, University of Nagasaki, Nagasaki, Japan; 2 Nutritional Epidemiology, Graduate School of Human Health Science, University of Nagasaki, Nagasaki, Japan; Hokkaido Daigaku, JAPAN

## Abstract

The circulating levels of β-carotene are modulated not only by sex, but also by autosomal gene variations and fruit intake. The aim of this study was to investigate the interactions between β-carotene metabolism-related gene single nucleotide polymorphisms (SNPs; genetic factors) and nutrient intake (environmental factors) relating to their effects on circulating β-carotene. The serum concentrations of β-carotene and the habitual food intake of 92 healthy Japanese adults were examined. All subjects were genotyped for three common SNPs: rs6564851 in the β-carotene 15,15′-oxygenase 1 (*BCO1*) gene, rs2278986 in the scavenger receptor class B member 1 (*SCARB1*) gene and rs362090 in the intestine-specific homeobox (*ISX*) gene. Univariate analysis revealed that the circulating β-carotene levels were significantly higher in rs6564851 GG homozygotes (*p* = 0.003). Additionally, the daily intake of β-cryptoxanthin was positively associated with the circulating β-carotene levels in female GG homozygotes of rs6564851 (*p* = 0.023), and the daily intake of α- and β-carotenes, and β-cryptoxanthin was significantly lower in female rs6564851 T allele carries than in female GG homozygotes (*p* = 0.009, 0.008, 0.009, respectively). The present study apparently indicates that higher circulating β-carotene levels in female rs6564851 GG homozygotes depend on carotenoid intake.

## Introduction

More than 25 years ago, Dr. Cutler found a significant positive correlation between the potential maximal lifespan of different primate species including humans and the concentration of carotenoids in their serum [1]. He also claimed that carotenoids may be biologically active as protective agents against cancer and as longevity determinants. Unlike retinol in the serum, circulating carotenoid levels in humans are well known to vary substantially among individuals [2–4], regions where the subjects live [5] and the season in which blood collection is performed [6], suggesting that circulating carotenoid levels depend on the amount of carotenoid intake prior to blood collection. Furthermore, oxidative stress such as smoking is known to decrease the circulating level of β-carotene [7]. As shown in the literature, the circulating level of β-carotene in humans is indeed influenced by several environmental factors.

In this context, a well-known observation of sex differences (higher in females than males) in circulating β-carotene levels has been explained by differences in the amount of fruit and vegetable intake [7]. In other words, higher circulating β-carotene and higher intake of dietary β-carotene in women compared with men have been repeatedly reported by many studies [7–12], however, we cannot exclude the possibility that the genetic background may also be involved in the sex differences in circulating β-carotene levels and the daily intake of carotenoids.

Recently, a genome-wide association study revealed that a common single-nucleotide polymorphism (SNP: rs6564851) near the β-carotene 15,15′-oxygenase (*BCO1*) gene affects the circulating levels of carotenoids in three Caucasian populations [13], suggesting that the genetic background, in addition to the above-mentioned environmental factors, may influence carotenoid levels in the blood. Therefore, gene-environment interaction studies on the circulating β-carotene level will provide new information that will help understand the physiological regulation of circulating carotenoid levels.

Transcription of the *BCO1* gene is known to be negatively regulated by the transcription factor, ISX (intestine specific homeobox), which binds to the 5′-upstream regulatory region of the *BCO1* gene [14]. Indeed, a mobility shift assay showed that recombinant ISX binds to a 21-bp synthetic oligonucleotide containing the above-mentioned common rs6564851 SNP site. Therefore, rs6564851 polymorphism may influence the binding affinity of ISX, although, Lobo et al. [14] have so far detected no difference in an *in vitro* reporter assay with the rs6564851 G/T alleles.

However, we cannot exclude the possibility that a putative *ISX* gene polymorphism is associated with *BCO1* gene expression. Although no association study has been reported on *ISX* gene polymorphism, we paid attention to a common nonsynonymous SNP (rs362090, Pro57Ser) at the N-terminal flanking region of the homeobox domain of ISX, which is registered in the NCBI SNP database. Hence, we were interested in examining whether “epistasis” exists between the *BCO1* and *ISX* genes, in which the *ISX* gene may be epistatic and the *BCO1* gene may be hypostatic or vice versa.

ISX protein binds to conserved DNA-binding motifs (5′-CTGGGATT-3′) upstream of the *BCO1* gene and the scavenger receptor class B member 1 (*SCARB1*) gene [15]. SCARB1 plays an important role in intestinal absorption of β-carotene into the epithelial cells [16], and the common intronic rs2278986 SNP in the *SCARB1* gene has been reported to be an independent predictor of SCARB1 protein levels and high-density lipoprotein cholesterol levels in subjects with hyperalphalipoproteinemia [17].

In the present study, we evaluated the effects of three SNPs (rs6564851 in *BCO1*, rs362090 in *ISX* and rs2278986 in *SCARB1*) and the dietary intake of carotenoids on serum carotenoid levels in 92 Japanese adult volunteers (63 men and 29 women).

## Subjects and Methods

### Study population

We enrolled 92 Japanese subjects, who were working in a town hall. Smokers and ex-smokers were excluded. Detailed information about diet and lifestyle habits, and a DNA sample from buccal cells, were collected from each participant, after they signed an informed consent form. The analyses were carried out on anonymously coded samples. The study plan was approved by the University Research Ethics Committee for Genome Research.

### Food frequency questionnaire (FFQ)

Data on habitual food intake were collected and processed using Excel *Eiyoukun*FFQ (Kenpakusha, Tokyo, Japan), which gave information about demographics, lifestyle and dietary intake. The FFQ comprised 115 food items that were divided into nine food groups of the same nutrient profile (cereals and cereal products, meat and meat products, fish and seafood, eggs, legumes and legume products, milk and milk products, vegetables and fruit, beverages, alcoholic beverages, and confectioneries, spreads and spices/miscellaneous items), with five categories of consumption frequency over 1 year. This commercially-available FFQ was validated by comparison with weighed dietary records for 7 continuous days (7d records) of 66 subjects aged 19–60 years [18].

### DNA preparation

To collect a buccal cell sample, the inside of both cheeks was firmly scraped three times with a Falcon^™^ single polyester fiber-tipped applicator swab (Becton Dickinson, East Rutherford, NJ). The swab was air-dried for at least 1 h after collection. DNA was extracted using the Qiagen Blood DNA Mini kit according to the manufacturer’s instructions (Qiagen K.K., Tokyo, Japan). The DNA preparations were frozen at −20°C until use.

### Genotyping

#### PCR-Restriction Fragment Length Polymorphism (RFLP)

Two common SNPs—rs6564851 near the *BCO1* gene and rs2278986 in the *SCARB1* gene—were genotyped by the PCR-RFLP method. The oligonucleotides used were: rs6564851, 5´-TTA TAT TGG CCT TGG CCG TTT C-3´ (forward primer) and 5´-AGG GAC CAT TCA AGG TTG TG-3´ (reverse primer); rs2278986, 5´-TAC ATC GTC ATG CCC AAC AT-3´ (forward primer) and 5´-CCA GGA CTT CCA AAC CAA GA-3´ (reverse primer). PCR reactions using Master Mix (Accupower, Bioneer, Daejeon, Korea) were run according to the manufacturer’s instructions. Thermal cycler conditions were as follows: initial melting at 94°C for 3 min, then 40 cycles of amplification at 95°C for 30 s, 56°C for 30 s (50°C for rs2278986), and 72°C for 90 s. This was followed by a final extension step of 72°C for 10 min. The product was then digested using BsrI or BsmAI (New England Biolabs, Boston, MA) (rs6564851 or rs2278986, respectively). The expected sizes of the digested products were examined by gel electrophoresis.

#### TaqMan PCR

rs362090 in the *ISX* gene was genotyped by PCR using TaqMan^®^ probes on an ABI PRISM 7300 Real-Time PCR System (Applied Biosystems, Life Technology Japan, Tokyo, Japan). Primers and probes were obtained from Applied Biosystems (Foster City, CA). TaqMan probes for allele A in rs362090 were labeled with FAM^™^ and those for allele G were labeled with VIC^™^. Automatic allele calling was selected to determine each genotype, according to the Applied Biosystems allele discrimination guide.

### Measurement of serum β-carotene

Tubes containing frozen serum were thawed and extracted within 30 min. A mixture of serum (100 μL) and ethanol (500 μL) was vortexed for 30 s, and then water (2 mL) and butylated hydroxytoluene with hexane (5 mL) were successively pipetted into 10-mL Pyrex tubes. After centrifugation at 1000 rpm for 10 min, the upper hexane phase (4 mL) was transferred into brown glass vials and dried under a nitrogen stream. The residue was dissolved in 100 μL of acetonitrile-dichloromethane-methanol (7:2:1 v/v/v), and 20 μL of the resultant solution were injected into a high-performance liquid chromatography (HPLC) column (5 μm, 250 × 4.6 mm, Mightysil RP-18 GP; Kanto Kagaku, Tokyo, Japan). The effluent was monitored for the detection of carotenoids using an ultraviolet-vis detector of SPD-20A (Shimadzu, Kyoto, Japan) equipped with Prominence HPLC apparatus (Shimadzu) at 450 nm. We used β-carotene type II, synthetic, ≥95% (HPLC), crystalline obtained from Sigma Aldrich Japan (Tokyo, Japan) as a standard and serum (stored frozen at -20°C) from one of us (MU) was used as a reference serum for inter-experimental control.

### Statistical analysis

The analyses were based on data reported by the dietary questionnaire administered at the beginning of the study. The influence of each dietary factor on the serum β-carotene concentration was estimated by logistic multivariate analysis adjusted for age, sex and energy intake. The difference in the serum β-carotene level between any two groups was parametrically evaluated by Student’s *t*-test. A correlation coefficient between the biomarkers investigated was calculated by Pearson correlation analysis. The level of statistical significance was set at *p* < 0.05. The SPSS/PC statistical software package (IBM, Tokyo, Japan) was used for the analyses.

## Results

### Allele frequencies of rs6564851, rs2278986 and rs362090

#### rs6564851

As shown in Table 1, in the present study the rs6564851 allele frequencies (G allele: 0.824 and T allele: 0.176), located near the *BCO1* gene, are consistent with those (0.833 and 0.167) in Japanese subjects in Tokyo (HapMap-JPT) in the NCBI SNP database (http://www.ncbi.nlm.nih.gov/projects/SNP/snp_ref.cgi?rs=6564851), whereas in Utah residents with Northern and Western European ancestry (HapMap-CEU) they are 0.467 and 0.533, and in Yoruba people in Ibadan, Nigeria (HapMap-YRI), they are 0.358 and 0.642, respectively.

**Table 1:** Genotype and allele frequencies of rs6564851 (*BCO1*), rs2278986 (*SCARB1*) and rs362090 (*ISX*) in each genotype group.

Gene	*BCO1*				*SCARB1*				*ISX*			
SNP	rs6564851				rs2278986				rs362090			
Genotype	GG	GT		TT	TT	TC		CC	AA	AG		GG
n	62	26		3	70	20		2	39	46		7
(M/F)	(40/22)	(19/7)		(3/0)	(51/19)	(12/8)		(0/2)	(31/8)	(28/18)		(4/3)
Frequency	0.681	0.286		0.033	0.0761	0.217		0.022	0.424	0.500		0.076
Allele	G		T		T		C		A		G	
Frequency	0.824		0.176		0.821		0.179		0.674		0.326	
(M/F)	(0.80/0.88)		(0.20/0.12)		(0.86/0.75)		(0.14/0.25)		(0.71/0.59)		(0.29/0.41)	
HWE*	p>0.05 (χ^2^ = 0.0193)				p>0.05 (χ^2^ = 3.1671)				p>0.05 (χ^2^ = 2.3343)			

* Hardy-Weinberg Equilibrium test was performed using Pearson's chi-squared test.

#### rs2278986

Table 1 shows that the rs2278986 allele frequencies (T allele: 0.821 and C allele: 0.179), one of the intronic SNPs in the *SCARB1* gene, are consistent with those (0.841 and 0.159) in HapMap-JPT in the NCBI SNP database, in which these values are 0.692 and 0.308 in European subjects in 1000 Genomes super population (EUR), and 0.759 and 0.241 in HapMap-YRI, respectively.

#### rs362090

Table 1 shows that the rs362090 allele frequencies (0.674 for the A allele and 0.326 for the G allele), a nonsynonymous SNP (Pro57Ser) of the *ISX* gene, are in the range of the frequencies (0.727 and 0.273) in HapMap-JPT in the NCBI SNP database, whereas the allele frequencies are 1.000 and 0.000 in the HapMap-CEU, and 0.559 and 0.441 in the HapMap-YRI population, respectively.

All allele polymorphism frequencies analyzed were in Hardy-Weinberg Equilibrium (HWE) in this population (*p* > 0.05; Table 1).

### Circulating β-carotene levels in each genotype group

Because the three polymorphisms tested herein are common SNPs with a minor allele frequency (MAF) of 0.05 or more, two groups of homozygotes carrying major alleles and the other minor-allele carriers were analyzed below for comparison (Table 2). The average concentrations of serum β-carotene in the rs6564851 GG homozygotes (the SNP of the *BCO1* gene) in total, males and females were significantly higher than those in the T allele carriers (*p* = 0.003, 0.039 and 0.027, respectively), showing that the GG homozygotes had a 2-fold higher serum β-carotene level compared with that of T allele carriers. In contrast, there was no significant difference in the serum β-carotene concentration between the major homozygotes and the minor allele carriers in the other two SNPs, rs2278986 (the SNP of the *SCARB1* gene) and rs362090 (the SNP of the *ISX* gene).

**Table 2:** Circulating β-carotene levels in each genotype group.

Genotype	n (M/F)	Total	Male	Female	
*BCO1* rs6564851	Serum β-carotene (μg/dL, mean ± SD)				*p* (Male vs. Female)
GG	59 (37/22)	63.9 ± 52.6	52.9 ± 54.7	82.4 ± 43.2	*0*.002**
GT;TT	27 (20/7)	31.2 ± 23.4	25.9 ± 20.9	46.2 ± 23.6	*0*.*056*
*p* (GG vs. T-carrier)		*0*.0032**	*0*.0394*	*0*.0271*	
*SCARB1* rs2278986					
TT	66 (47/19)	53.8 ± 50.5	46.1 ± 50.3	73.0 ± 45.8	*0*.004**
TC;CC	21 (11/10)	52.2 ± 37.7	31.6 ± 27.1	74.9 ± 34.6	*0*.004**
*p* (TT vs. C-carrier)		*0*.*6648*	*0*.*6882*	*0*.*7699*	
*ISX* rs362090					
AA	35 (27/8)	57.4 ± 51.9	49.2 ± 52.9	85.1 ± 28.7	*0*.030*
AG;GG	52 (31/21)	50.8 ± 44.5	38.3 ± 40.8	69.3 ± 43.4	*0*.001**
*p* (AA vs. G-carrier)		*0*.*6815*	*0*.*3659*	*0*.*3454*	

*: Significantly different by gender or genotype group, analyzed using the Student’s t-test (*: *p* < 0.05)

**: Significantly different by gender or genotype group, analyzed using the Student’s t-test (**: *p* < 0.01)

As shown in Table 2, there were significant differences in the average concentrations of serum β-carotene between males and females of any genotype group, except for the rs6564851 T allele carriers (*p* = 0.056).

### Daily intake of fruit and carotenoids in each genotype group

As the rs6564851 GG homozygotes (SNP of the *BCO1* gene) showed a higher circulating level of β-carotene in total, males and females than the T allele carriers, we next surveyed their daily intake of carotenoid-containing foods. Table 3 clearly shows that the daily intake of fruit, α-carotene, β-carotene, β-cryptoxanthin and β-carotene equivalent by female rs6564851 T allele carriers was significantly lower than that by female GG homozygotes. However, in males there was no significant difference in the daily intake of these foods and nutrients between the T allele carriers and GG homozygotes. Furthermore, we found no significant differences in the daily intake of energy, protein, fat, carbohydrates, sodium and other minerals, vitamin D, α-tocopherol, and all species of fatty acids, between female GG homozygotes and T allele carriers, except for the daily intake of vitamin C by female T allele carriers, which was lower than that by female GG homozygotes (*p* = 0.009, see S1 Table).

**Table 3:** Daily intake of fruit and carotenoids of males and females in each genotype group.

	Male			Female		
Daily intake	rs6564851 (*BCO1*)					
(mean ±SD) of	GG	GT;TT	*p*	GG	GT;TT	*p*
fruit (g)	44 ± 42	46 ± 46	*0*.*4821*	51 ± 41	14 ± 15	*0*.0079**
retinol (μg)	201 ± 58	190 ± 86	*0*.*6154*	197 ± 47	167 ± 56	*0*.*1519*
α-carotene (μg)	349 ± 182	339 ± 221	*0*.*8700*	487 ± 230	259 ± 225	*0*.0091**
β-carotene (μg)	2119 ± 1078	2063 ± 1318	*0*.*8499*	2948 ± 1356	1553 ± 1324	*0*.0076**
β-cryptoxanthin (μg)	297 ± 250	304 ± 276	*0*.*4989*	331 ± 241	107 ± 99	*0*.0090**
β-carotene equiv. (μg)	2449 ± 1212	2390 ± 1506	*0*.*9028*	3364 ± 1529	1746 ± 1478	*0*.0063**
	rs2278986 (*SCARB1*)					
	TT	TC;CC	*p*	TT	TC;CC	*p*
fruit (g)	39 ± 40	62 ± 61	*0*.*3350*	34 ± 33	57 ± 46	*0*.*1036*
retinol (μg)	205 ± 72	211 ± 56	*0*.*7351*	190 ± 53	189 ± 47	*0*.*9774*
α-carotene (μg)	349 ± 191	391 ± 210	*0*.*9486*	400 ± 219	493 ± 287	*0*.*3538*
β-carotene (μg)	2118 ± 1132	2384 ± 1258	*0*.*4923*	2427 ± 1302	2963 ± 1702	*0*.*3688*
β-cryptoxanthin (μg)	273 ± 234	403 ± 367	*0*.*3371*	231 ± 196	365 ± 277	*0*.*1334*
β-carotene equiv. (μg)	2437 ± 1267	2788 ± 1471	*0*.*5931*	2750 ± 1461	3398 ± 1932	*0*.*3419*
	rs362090 (*ISX*)					
	AA	AG;GG	*p*	AA	AG;GG	*p*
fruit (g)	38 ± 41	49 ± 48	*0*.*4389*	54 ± 36	37 ± 40	*0*.*1966*
retinol (μg)	201 ± 58	190 ± 86	*0*.*6154*	197 ± 47	167 ± 56	*0*.*1519*
α-carotene (μg)	341 ± 163	373 ± 221	*0*.*8036*	500 ± 289	406 ± 233	*0*.*3653*
β-carotene (μg)	2073 ± 959	2261 ± 1322	*0*.*7849*	3027 ± 1671	2453 ± 1396	*0*.*3445*
β-cryptoxanthin (μg)	263 ± 244	332 ± 289	*0*.*3909*	349 ± 214	249 ± 238	*0*.*2212*
β-carotene equiv. (μg)	2383 ± 1062	2620 ± 1512	*0*.*7326*	3459 ± 1837	2788 ± 1594	*0*.*3226*

**: Significantly different by genotype groups, analyzed using the Student’s t-test (*p* < 0.01)

There was no significant difference in the daily intake of fruit and carotenoid-related nutrients between major allele homozygotes and minor allele carriers of rs2278986 (*SCARB1*) and rs362090 (*ISX*) (Table 3).

### Association of the circulating β-carotene level with the daily intake of fruit and carotenoid-related nutrients in rs6564851 GG homozygotes (*BCO1*)

Next, we analyzed the association of the serum β-carotene concentration with environmental factors, especially the daily intake of carotenoid-related nutrients. A statistically significant association of the circulating level of β-carotene was detected with the daily intake of β-cryptoxanthin (*p* < 0.05) and vitamin C (*p* < 0.05), and a stronger association was found with the daily intake of β-cryptoxanthin (*p* < 0.01) in the rs6564851 GG homozygotes. However, no association of the serum β-carotene concentration with the daily intake of carotenoid-related nutrients was detected in the T allele carriers (Table 4). These significant associations may be explained by the daily intake of carotenoid-containing foods by the GG homozygotes (see S2 Table). In contrast, the association of the circulating level of β-carotene was strongly associated with the daily intake of β-cryptoxanthin in females (*p* < 0.01), whereas males did not show any association (Table 4).

**Table 4:** Association of circulating β-carotene level with daily intake of nutrients.

Genotype	n	Retinol		α-Carotene		β-Carotene		β-Cryptoxanthin		β-Carotene equivalent	
All											
rs6564851											
GG	59	0.248	(0.058)	0.236	(0.072)	0.244	(0.062)	0.342**	(0.008)	0.269*	(0.039)
GT/TT	27	-0.045	(0.822)	-0.060	(0.766)	-0.064	(0.750)	-0.023	(0.908)	-0.060	(0.767)
Both	86	0.108	(0.317)	0.157	(0.147)	0.162	(0.134)	0.250*	(0.020)	0.180	(0.095)
Male											
rs6564851											
GG	37	0.228	(0.175)	0.019	(0.911)	0.025	(0.883)	0.252	(0.133)	0.055	(0.747)
GT/TT	20	0.161	(0.498)	0.057	(0.812)	0.059	(0.806)	0.106	(0.658)	0.066	(0.781)
Both	57	0.105	(0.435)	-0.007	(0.959)	-0.005	(0.972)	0.194	(0.144)	0.018	(0.892)
Female											
rs6564851											
GG	22	0.003	(0.988)	0.227	(0.310)	0.235	(0.293)	0.483*	(0.023)	0.271	(0.223)
GT/TT	7	-0.388	(0.377)	0.023	(0.961)	0.023	(0.961)	-0.006	(0.991)	0.037	(0.937)
Both	29	0.025	(0.898)	0.334	(0.077)	0.342	(0.069)	0.517**	(0.004)	0.369	(0.049)

Data are shown as *r* (*p*-trend), analyzed using the Pearson’s correlation coefficient test after logarithmic transformation of circulating β-carotene level and square-root transformation of daily intake of nutrients,

* *p* < 0.05,

** *p* < 0.01.

## Discussion

It is well known that circulating levels of β-carotene are highly variable among individuals, regions and seasons, which has so far been explained by many factors: 1) fruit and vegetable intake, 2) sex, 3) oxidative stress such as smoking and exercise, 4) aging, and 5) genetic background. In the present study on a healthy adult Japanese population, we confirmed two previous findings: the serum concentration of β-carotene is higher in females than in males [4], and a regulatory SNP (rs6564851) of the *BCO1* gene significantly affects the circulating levels of β-carotene, which were higher in the GG homozygotes than in the T allele carriers [13]. Furthermore, we found that compared with female GG homozygotes, female rs6564851 T allele carriers showed a significantly lower consumption of fruit and a lower intake of carotenoids such as α- and β-carotene, β-cryptoxanthin and β-carotene equivalents.

Most epidemiological studies have repeatedly reported that the plasma concentrations of β-carotene are positively associated with the daily intake of fruit and vegetables [4,5,19,20], indicating plasma β-carotene as a biomarker of vegetable and fruit intake. This may explain the seasonal variations in plasma β-carotene concentrations, which are high in the summer and low in the winter [21,22]. In the present study, the blood samples were obtained in the summer, hence the circulating levels of β-carotene may be higher than in other seasons. Nevertheless, we did not detect any association between the circulating β-carotene level and the daily intake of green/yellow vegetables, other vegetables and fruit (S2 Table). Also, there was no significant association between the circulating β-carotene level and the daily intake of α- and β-carotene, or β-carotene equivalent, except for β-cryptoxanthin, although a weak association was found with the daily intake of β-carotene (Table 4). Consistent with our findings, Jansen et al. who investigated the relationship between plasma carotenoids and the usual vegetable and fruit intake, have reported that the plasma β-carotene concentration could not distinguish quartiles of vegetables, fruit and/or juice intake assessed by FFQ [23]. In general, the FFQ measures the daily food intake by asking about the consumption frequency over the past year. Thus, the resulting data of the FFQ do not directly reflect the food intake immediately before blood sampling, which may be one of the reasons for not detecting an association between the circulating β-carotene level and the daily intake of green/yellow vegetables, other vegetables and fruit.

As another possibility, we should discuss the sample size as a possible limitation of the study design, and the possibility that our study was underpowered to detect the expected association between β-carotene intake and circulating β-carotene levels. This possibility is underscored by the marginal *p*-value observed for the association between β-carotene intake and circulating β-carotene levels in GG homozygotes, which was 0.062 (Table 4). We speculate that with a larger sample size, a significant association may have been observed.

However, we were able to detect a significant positive association between the circulating β-carotene level and the daily intake of β-carotene equivalent in the rs6564851 GG homozygotes (*p* = 0.039, Table 4), who are expected to express a lower level of BCO1. As described in the Introduction, a genome-wide association study with three different cohorts [13] has revealed that the GG homozygotes showed higher circulating levels of β-carotene than the T allele carriers. Moreover, *in vivo* studies supportively revealed that the catalytic activity of BCO1 was reduced by 48% in female GG homozygotes and the retinyl palmitate:β-carotene ratio in the TG-rich lipoprotein fraction positively correlated with the rs6564851 T allele, indicating that the GG homozygote is a poor responder to conversion of β-carotene to retinyl esters through retinal and retinol [24,25]. In this context, it is easy to understand that a significant positive association of the circulating β-carotene level with the daily intake of β-carotene equivalent was found in the GG homozygotes, by speculating that in the GG homozygotes the ingested β-carotene may efficiently enter the blood stream without excessive cleavage by BCO1 activity. Consistently, it is understandable that the circulating β-carotene level in the T allele carriers, who are expected to express a higher level of BCO1, showed no association with the daily intake of fruit, α- and β-carotene, β-cryptoxanthin and β-carotene equivalent, because in the T allele carriers the ingested β-carotene may be efficiently converted to retinoids by intestinal BCO1 without excessive increment of the circulating β-carotene level.

Although a sample size of female rs6564851 T allele carriers is very small, one of the most interesting findings of the present study is that female rs6564851 T allele carriers eat significantly less fruit, and their daily intake of α- and β-carotene, β-cryptoxanthin, and β-carotene equivalent is dramatically lower than that of the GG homozygotes, suggesting that the *BCO1* genotype (GG or T allele carrier) may influence eating behavior of fruit and carotenoids, but not of other micro- (e.g., vitamin D and α-tocopherol) and macro-nutrients (e.g., protein, fat and carbohydrates), minerals (sodium, calcium and iron) and energy (see S1 Table). In general, food preference is thought to be dependent on taste sensing, however, it is very hard to speculate that the *BCO1* genotype is involved in producing inter-individual differences in taste sensing of carotenoids. But, to explain why the carotenoid preference differs by the *BCO1* genotype, we are wondering a highly speculative hypothesis that postoral sensing [26] of carotenoids such as a putative “gut β-carotene sensing by BCO1” may exist and comprise a food rewarding system in the absence of taste receptor signaling [27].

As another, more feasible and more important idea, vitamin A metabolised from the ingested β-carotene may play a role in controlling eating behavior. Female T-allele carriers consume less fruit because they are efficient cleavers of β-carotene and their body signals them to consume less fruit because they have all the required nutrients. On other hand, GG homozygotes consume excess fruit because they are inefficient cleavers and must eat more fruit to get the requisite amount of vitamin A their body needs.

As for the effect of the genetic background on serum β-carotene concentrations, other than rs6564851, a couple of common nonsynonymous SNPs (Ala379Val: rs7501331, Arg267Ser: rs12934922) of the *BCO1* gene have been reported to be associated with the circulating levels of β-carotene [28]. Indeed, *in vitro* biochemical characterization of the recombinant 267Ser + 379Val double mutant revealed a reduced catalytic activity of BCO1 by 57%. Furthermore, assessment of the responsiveness of female volunteers to a pharmacological dose of β-carotene confirmed that carriers of both the 379Val and 267Ser + 379Val variant alleles had a reduced ability to convert β-carotene and increased fasting β-carotene concentrations [28]. This study clearly explained the existence of a poor responder phenotype of β-carotene conversion to retinyl esters through retinal and retinol upon carotenoid loading. However, meta-analyses with a genome-wide association study revealed that the rs6564851 G allele, near the *BCO1* gene, rather than any common SNP residing inside of the *BCO1* gene, was strongly associated with higher circulating β-carotene and α-carotene levels, and lower circulating lycopene, zeaxanthin and lutein levels [13]. Lietz et al., who reported the poor responder SNPs, found that the retinyl palmitate:β-carotene ratio in the TG-rich lipoprotein fraction positively correlated with the rs6564851 T allele in healthy female volunteers, and that the fasting plasma β-carotene concentration positively correlated with the rs11645428 G allele, whereas it negatively correlated with the rs6564851 T allele [24]. Therefore, we decided to focus on the rs6564851 SNP in our study, to investigate the effect of genetic background on the circulating β-carotene levels.

Recently, we have reported that high dietary intake of the antioxidants, α and β-carotene, and α-tocopherol, protects buccal cells from relative telomere length (RTL) shortening, depending on the genetic background of the *BCO1* and *ISX* genes, with the same population analyzed in the present study [29]. In brief, we showed a positive effect of daily α- or β-carotene intake on buccal RTL only in the *ISX* rs362090 G allele carrier + *BCO1* rs6564851 GG genotype group. Because we found an apparent interactive effect of the *ISX* gene SNP rs362090 on the *BCO1* rs6564851, we analyzed the effect of rs362090 and rs2278986 in the *SCARB1* gene (one of the target genes of ISX) on the circulating β-carotene level and daily intake of carotenoid-containing foods. We were unable to detect any effects of these SNPs on the circulating level of β-carotene or on the daily intake of carotenoid-containing foods.

As mentioned above, there was a unique association between the circulating β-carotene level and the daily intake of β-cryptoxanthin. In particular, females showed a strong positive association with β-cryptoxanthin intake. Currently, we cannot explain why the serum β-carotene concentration associated with the daily intake of β-cryptoxanthin, but not with β-carotene. Burri has claimed that β-cryptoxanthin has greater bioavailability from its common food sources than do α- and β-carotene [30]. Although β-cryptoxanthin appears to be a poorer substrate for BCO1 than β-carotene, animal models and human studies suggest that the comparatively high bioavailability of β-cryptoxanthin from foods makes β-cryptoxanthin-rich foods equivalent to β-carotene-rich foods as sources of vitamin A [30]. Indeed, eccentric cleavage by the mitochondrial β-carotene-9′,10′-oxygenase (BCO2) converts β-cryptoxanthin to vitamin A [31], and then retinoic acid induces cellular expression of ISX, which suppresses transcription of the *BCO1* gene [15], resulting in upregulation of the circulating β-carotene level [31]. Ingested β-cryptoxanthin may be a major negative regulator of *BCO1* gene expression.

It may be also worthwhile to discuss about a possible negative regulator of BCO1 enzyme activity. Lycopene can be a competitive inhibitor against β-carotene cleavage, because lycopene is a best affinity substrate among carotenoids in ordinary foods, its *K*m value (1.7 μM) is one tenth of that (17 μM) for β-carotene [32]. So, it is apparently reasonable to speculate that lycopene intake may competitively perturb BCO1-mediated cleavage of dietary β-carotene in intestine, which may result in upregulation of circulating β-carotene level. Unfortunately, however, lycopene intake was not measured in the present study, because lycopene analysis lacks in the FFQ application software we used.

In any event, before drawing firm conclusions, these findings should be reproduced by other studies using designs that avoid biases from sample type, sample size, and assay method.

In conclusion, we demonstrated here that the common SNP, rs6564851 near the *BCO1* gene, affects the circulating levels of β-carotene and the daily intake of carotenoids in healthy Japanese women, which is consistent with previous findings by genome-wide association study in 3 Caucasian populations [13]. We further showed the interactive effects of genetic factors (sex and rs6564851) and environmental factors (daily intake of fruit, β-cryptoxanthin and β-carotene equivalent) on the circulating levels of β-carotene in healthy Japanese adults.

## Supporting Information

S1 Table Daily intakes of energy and other nutrients by males and females in rs6564851 genotype groups. **: Significantly different by genotype groups, analyzed using the Student’s t-test (p < 0.01).
(DOCX)

S2 Table Association of circulating β-carotene level with daily intake of carotenoid-containing foods. Data are shown as r (p-trend), analyzed using Pearson’s correlation coefficient test after logarithmic transformation of circulating β-carotene level and square-root transformation of daily intake of foods, *p < 0.05.
(DOCX)

## References

[1] Cutler RG. Carotenoids and retinol: their possible importance in determining longevity of primate species. Proc Natl Acad Sci U S A. 1984;81: 7627–31. Available: http://www.pubmedcentral.nih.gov/articlerender.fcgi?artid=392201&tool=pmcentrez&rendertype=abstract 6594706 10.1073/pnas.81.23.7627 PMC392201

[2] Parker RS. Carotenoids in human blood and tissues. J Nutr. 1989;119: 101–4. Available: http://www.ncbi.nlm.nih.gov/pubmed/2643690 2643690 10.1093/jn/119.1.101

[3] Tangney CC, Shekelle RB, Raynor W, Gale M, Betz EP. Intra- and interindividual variation in measurements of?? -carotene, retinol, and tocopherols in diet and plasma. Am J Clin Nutr. 1987;45: 764–769. 3565304 10.1093/ajcn/45.4.764

[4] Al-Delaimy WK, Ferrari P, Slimani N, Pala V, Johansson I, Nilsson S, et al Plasma carotenoids as biomarkers of intake of fruits and vegetables: individual-level correlations in the European Prospective Investigation into Cancer and Nutrition (EPIC). Eur J Clin Nutr. 2005;59: 1387–96. 10.1038/sj.ejcn.1602252 16160702

[5] Al-Delaimy WK, Slimani N, Ferrari P, Key T, Spencer E, Johansson I, et al Plasma carotenoids as biomarkers of intake of fruits and vegetables: ecological-level correlations in the European Prospective Investigation into Cancer and Nutrition (EPIC). Eur J Clin Nutr. 2005;59: 1397–1408. 10.1038/sj.ejcn.1602253 16160701

[6] Siegel EM, Craft NE, Roe DJ, Duarte-Franco E, Villa LL, Franco EL, et al Temporal variation and identification of factors associated with endogenous retinoic acid isomers in serum from Brazilian women. Cancer Epidemiol Biomarkers Prev. 2004;13: 1693–703. Available: http://www.ncbi.nlm.nih.gov/pubmed/15533895 15533895

[7] Brady WE, Mares-Perlman JA, Bowen P, Stacewicz-sapuntzakis M. Human serum carotenoids concentrations are related to physiologic and lifestyle factors. J Nutr. 1996;126: 129–137. 8558292 10.1093/jn/126.1.129

[8] Kritchevsky SB, Bush AJ, Pahor M, Gross MD. Serum carotenoids and markers of inflammation in nonsmokers. Am J Epidemiol. 2000;152: 1065–1071. 11117616 10.1093/aje/152.11.1065

[9] Ford ES, Liu S, Mannino DM, Giles WH, Smith SJ. C-reactive protein concentration and concentrations of blood vitamins, carotenoids, and selenium among United States adults. Eur J Clin Nutr. 2003;57: 1157–63. 10.1038/sj.ejcn.1601667 12947436

[10] Gruber M, Chappell R, Millen A, LaRowe T, Moeller SM, Iannaccone A, et al Correlates of serum lutein + zeaxanthin: findings from the Third National Health and Nutrition Examination Survey. J Nutr. 2004;134: 2387–94. Available: http://www.ncbi.nlm.nih.gov/pubmed/15333733 15333733 10.1093/jn/134.9.2387

[11] Wang L, Gaziano JM, Norkus EP, Buring JE, Sesso HD. Associations of plasma carotenoids with risk factors and biomarkers related to cardiovascular disease in middle-aged and older women. Am J Clin Nutr. 2008;88: 747–754. 18779292 10.1093/ajcn/88.3.747 PMC2559966

[12] Rydén M, Garvin P, Kristenson M, Leanderson P, Ernerudh J, Jonasson L. Provitamin a carotenoids are independently associated with matrix metalloproteinase-9 in plasma samples from a general population. J Intern Med. 2012;272: 371–384. 10.1111/j.1365-2796.2012.2534.x 22486775

[13] Ferrucci L, Perry JRB, Matteini A, Perola M, Tanaka T, Silander K, et al Common Variation in the beta-Carotene 15,15′-Monooxygenase 1 Gene Affects Circulating Levels of Carotenoids: A Genome-wide Association Study. Am J Hum Genet. 2009;84: 123–133. 10.1016/j.ajhg.2008.12.019 19185284 PMC2668002

[14] Lobo GP, Amengual J, Baus D, Shivdasani RA, Taylor D, Von Lintig J. Genetics and diet regulate vitamin A production via the homeobox transcription factor ISX. J Biol Chem. 2013;288: 9017–9027. 10.1074/jbc.M112.444240 23393141 PMC3610974

[15] Widjaja-Adhi MAK, Lobo GP, Golczak M, Von Lintig J. A genetic dissection of intestinal fat-soluble vitamin and carotenoid absorption. Hum Mol Genet. 2015;24: 3206–19. 10.1093/hmg/ddv072 25701869 PMC4424956

[16] Lobo GP, Hessel S, Eichinger A, Noy N, Moise AR, Wyss A, et al ISX is a retinoic acid-sensitive gatekeeper that controls intestinal beta,beta-carotene absorption and vitamin A production. FASEB J. 2010;24: 1656–66. 10.1096/fj.09-150995 20061533 PMC2874479

[17] West M, Greason E, Kolmakova A, Jahangiri A, Asztalos B, Pollin TI, et al Scavenger receptor class B type I protein as an independent predictor of high-density lipoprotein cholesterol levels in subjects with hyperalphalipoproteinemia. J Clin Endocrinol Metab. 2009;94: 1451–1457. 10.1210/jc.2008-1223 19158204 PMC2682469

[18] Takahashi K, Yoshimura Y, Kaimoto T, Kunii D, Komatsu T. Validation of a Food Frequency Questionnaire Based on Food Groups for Estimating Individual Nutrient Intake. Japanese J Nutr Diet. 2001;59: 221–232.

[19] Campbell DR, Gross MD, Martini MC, Grandits GA, Slavin JL, Potter JD. Plasma carotenoids as biomarkers of vegetable and fruit intake. Cancer Epidemiol Biomarkers Prev. 1994;3: 493–500. 8000300

[20] Higuchi K, Saito I, Maruyama K, Eguchi E, Mori H, Tanno S, et al Associations of serum β-carotene and retinol concentrations with insulin resistance: The Toon Health Study. Nutrition. Elsevier Inc.; 2015;31: 975–980. 10.1016/j.nut.2015.02.015 26059371

[21] Xiang J, Nagaya T, Huang XE, Kuriki K, Imaeda N, Tokudome Y, et al Sex and seasonal variations of plasma retinol,?? -tocopherol, and carotenoid concentrations in Japanese dietitians. Asian Pacific J Cancer Prev. 2008;9: 413–416. 18990012

[22] Kolodziejczyk JK, Flatt SW, Natarajan L, Patterson R, Pierce JP, Norman GJ. Associations of soluble fiber, whole fruits/vegetables, and juice with plasma Beta-carotene concentrations in a free-living population of breast cancer survivors. Women Health. 2012;52: 731–43. 10.1080/03630242.2012.728189 23127215 PMC3491579

[23] Jansen MCJF, Van Kappel A L, Ocké MC, Van ‘t Veer P, Boshuizen HC, Riboli E, et al Plasma carotenoid levels in Dutch men and women, and the relation with vegetable and fruit consumption. Eur J Clin Nutr. 2004;58: 1386–1395. 10.1038/sj.ejcn.1601981 15054421

[24] Lietz G, Oxley A, Leung W, Hesketh J. Single nucleotide polymorphisms upstream from the β-carotene 15,15’-monoxygenase gene influence provitamin A conversion efficiency in female volunteers. J Nutr. 2012;142: 161S–5S. 10.3945/jn.111.140756 22113863

[25] Feigl B, Morris CP, Voisey J, Kwan A, Zele AJ. The relationship between BCMO1 gene variants and macular pigment optical density in persons with and without age-related macular degeneration. PLoS One. 2014;9. 10.1371/journal.pone.0089069 PMC3929644 24586510

[26] Sclafani A, Ackroff K. Role of gut nutrient sensing in stimulating appetite and conditioning food preferences. Am J Physiol Regul Integr Comp Physiol. 2012;302: R1119–33. 10.1152/ajpregu.00038.2012 22442194 PMC3362145

[27] de Araujo IE, Oliveira-Maia AJ, Sotnikova TD, Gainetdinov RR, Caron MG, Nicolelis MAL, et al Food Reward in the Absence of Taste Receptor Signaling. Neuron. 2008;57: 930–941. 10.1016/j.neuron.2008.01.032 18367093

[28] Leung WC, Hessel S, Méplan C, Flint J, Oberhauser V, Tourniaire F, et al Two common single nucleotide polymorphisms in the gene encoding beta-carotene 15,15’-monoxygenase alter beta-carotene metabolism in female volunteers. FASEB J. 2009;23: 1041–1053. 10.1096/fj.08-121962 19103647

[29] Yabuta S, Masaki M, Shidoji Y. Associations of buccal cell telomere length with daily intake of β-carotene or α-tocopherol are dependent on carotenoid metabolism-related gene polymorphisms in healthy Japanese adults. J Nutr Heal Aging. 2016;20: 267–274. 10.1007/s12603-015-0577-x PMC12877614 26892575

[30] Burri BJ. . Beta-cryptoxanthin as a source of vitamin A. J Sci Food Agric. 2015;95: 1786–1794. 10.1002/jsfa.6942 25270992

[31] Amengual J, Widjaja-Adhi MAK, Rodriguez-Santiago S, Hessel S, Golczak M, Palczewski K, et al Two carotenoid oxygenases contribute to Mammalian provitamin a metabolism. J Biol Chem. 2013;288: 34081–34096. 10.1074/jbc.M113.501049 24106281 PMC3837149

[32] Dela Seña C, Narayanasamy S, Riedl KM, Curley RW, Schwartz SJ, Harrison EH. Substrate specificity of purified recombinant human β-carotene 15,15′-oxygenase (BCO1). J Biol Chem. 2013;288: 37094–37103. 10.1074/jbc.M113.507160 24187135 PMC3873565

