# Supplementary material for: Common SNP rs6564851 in the BCO1 Gene Affects the Circulating Levels of β-Carotene and the Daily Intake of Carotenoids in Healthy Japanese Women
Source: PLoS One. 2016 Dec 22;11(12):e0168857. doi: 10.1371/journal.pone.0168857 (PMC5179075; doi:10.1371/journal.pone.0168857)
Supplement: S1 Table — **: Significantly different by genotype groups, analyzed using the Student’s t-test (p < 0.01). (DOCX) [file pone.0168857.s001.docx]

**S1 Table. Daily intakes of energy and other nutrients by males and females in rs6564851 genotype groups**

| Daily Intake (mean ± SD) of | **Male** | | | **Female** | | |
| --- | --- | --- | --- | --- | --- | --- |
|  | GG | GT; TT | *p* | GG | GT; TT | *p* |
| Energy (kcal) | 1990 ± 362 | 1478 ± 582 | *0.0728* | 1676 ± 265 | 1579 ± 336 | *0.4529* |
| Protein (g) | 66.2 ± 15.6 | 54.2 ± 24.5 | *0.5514* | 62.2 ± 11.4 | 50.7 ± 20.5 | *0.0816* |
| Fat (g) | 65.2 ± 15.9 | 50.0 ± 22.7 | *0.8511* | 61.2 ± 13.7 | 56.1 ± 15.6 | *0.4323* |
| Carbohydrate (g) | 253.5 ± 59.6 | 186.7 ± 76.9 | *0.0074* | 208.2 ± 44.3 | 208.6 ± 28.2 | *0.9846* |
|  |  |  |  |  |  |  |
| Sodium (mg) | 3705 ± 1117 | 2698 ± 1066 | *0.0369* | 3430 ± 1502 | 2586 ± 794 | *0.1807* |
| Calcium (mg) | 483 ± 147 | 473 ± 219 | *0.6490* | 464 ± 110 | 395 ± 152 | *0.2191* |
| Iron (mg) | 6.9 ± 1.8 | 5.2 ± 2.7 | *0.2637* | 6.4 ± 1.4 | 5.6 ± 2.5 | *0.2945* |
| Vitamin D (μg) | 8.9 ± 1.0 | 9.4 ± 1.5 | *0.3903* | 8.7 ± 0.7 | 8.2 ± 0.5 | *0.0989* |
| α-Tocopherol (mg) | 11.0 ± 1.4 | 9.9 ± 1.4 | *0.8414* | 11.0 ± 1.4 | 10.6 ± 1.0 | *0.4934* |
| Vitamin C (mg) | 58 ± 22 | 56 ± 33 | *0.8399* | 72 ± 27 | 39 ± 23 | *0.0091*** |
| Saturated fatty acid (g) | 19.4 ± 5.6 | 16.7 ± 7.3 | *0.9672* | 19.0 ± 4.7 | 16.6 ± 5.4 | *0.2720* |
| Mono unsaturated fatty acid (g) | 23.3 ± 5.9 | 17.5 ± 8.2 | *0.8633* | 22.1 ± 5.5 | 19.3 ± 6.3 | *0.2921* |
| Poly unsaturated fatty acid (g) | 13.3 ± 3.3 | 9.6 ± 4.3 | *0.5151* | 12.2 ± 3.2 | 11.1 ± 3.5 | *0.4718* |
| Cholesterol (mg) | 325 ± 79 | 275 ± 143 | *0.7512* | 299 ± 72 | 271 ± 120 | *0.4764* |
