# Supplementary material for: Common SNP rs6564851 in the BCO1 Gene Affects the Circulating Levels of β-Carotene and the Daily Intake of Carotenoids in Healthy Japanese Women
Source: PLoS One. 2016 Dec 22;11(12):e0168857. doi: 10.1371/journal.pone.0168857 (PMC5179075; doi:10.1371/journal.pone.0168857)
Supplement: S2 Table — Data are shown as r (p-trend), analyzed using Pearson’s correlation coefficient test after logarithmic transformation of circulating β-carotene level and square-root transformation of daily intake of foods, *p < 0.05. (DOCX) [file pone.0168857.s002.docx]

**S2 Table. Association of circulating β-carotene level with daily intake of carotenoid-containing foods**

| Genotypes | n | Green yellow vegetables | | Other vegetables | | Fruits | |
| --- | --- | --- | --- | --- | --- | --- | --- |
| All  rs6564851 |  |  |  |  |  |  |  |
| GG | 59 | 0.202 | (0.126) | 0.135 | (0.307) | 0.313 | (0.016) |
| GT/TT | 27 | -0.044 | (0.829) | -0.012 | (0.952) | 0.038 | (0.852) |
| Both | 86 | 0.025 | (0.898) | 0.334 | (0.077) | 0.342 | (0.069) |
| Male  rs6564851 |  |  |  |  |  |  |  |
| GG | 37 | 0.011 | (0.946) | -0.121 | (0.474) | 0.204 | (0.225) |
| GT/TT | 20 | 0.070 | (0.770) | 0.245 | (0.298) | 0.157 | (0.510) |
| Both | 57 | -0.003 | (0.985) | -0.049 | (0.717) | 0.189 | (0.156) |
| Female  rs6564851 |  |  |  |  |  |  |  |
| GG | 22 | 0.224 | (0.316) | 0.129 | (0.568) | 0.459 | (0.032) |
| GT/TT | 7 | 0.024 | (0.960) | -0.187 | (0.688) | -0.051 | (0.913) |
| Both | 29 | 0.338 | (0.340) | 0.227 | (0.529) | 0.528 | (0.117) |
